# Murray law-based quantitative flow ratio versus invasive fractional flow reserve in the left anterior descending coronary artery: impact of hydrostatic pressure correction and clinical implications

**DOI:** 10.3389/fcvm.2026.1806501

**Published:** 2026-06-17

**Authors:** András Ágoston, Ádám Piricsi, Bettina Szekeres, Mátyás Magyari, Balázs Tar, Gábor T. Szabó, Csaba A. Dézsi, Zsolt Piroth, Zoltán Ruzsa, Gábor G. Tóth, Shengxian Tu, Zsolt Kőszegi

**Affiliations:** 1 Department of Cardiology, Szabolcs-Szatmár-Bereg Country Hospitals and University Teaching Hospital, Nyíregyháza, Hungary; 2 Kálmán Laki Doctoral School of Biomedical and Clinical Sciences, University of Debrecen, Debrecen, Hungary; 3 Division of Cardiology, Department of Cardiology, Faculty of Medicine, University of Debrecen, Debrecen, Hungary; 4 Center for Biomedical Research and Translational Surgery, Medical University of Vienna, Vienna, Austria; 5 Department of Cardiology, Petz Aladár University Teaching Hospital, Győr, Hungary; 6 Faculty of Health and Sport Sciences, Széchenyi István University, Győr, Hungary; 7 Gottsegen National Cardiovascular Center, Budapest, Hungary; 8 Cardiology Center, Invasive Cardiology Unit, University of Szeged, Szeged, Hungary; 9 Division of Cardiology, Medical University of Graz, Graz, Austria; 10 School of Biomedical Engineering, Shanghai Jiao Tong University, Shanghai, China

**Keywords:** fractional flow reserve (FFR), hydrostatic pressure (HP), left anterior descending artery (LAD), Murray law-based quantitative flow ratio (μQFR), quantitative flow ratio (QFR)

## Abstract

**Background:**

Invasive fractional flow reserve (FFR) is the gold standard for guiding coronary revascularization. Angiography-derived FFR techniques such as the quantitative flow ratio (QFR) provide a less invasive alternative. Prior studies generally demonstrated good agreement between QFR and FFR, but the effect of hydrostatic pressure differences has not been quantitated as a potential source of systematic discrepancy. We investigated whether correcting invasive FFR for hydrostatic pressure error improved its agreement with QFR in the left anterior descending artery (LAD) lesions.

**Methods:**

We studied 33 coronary lesions in the LAD. Invasive FFR was measured using the standard pressure-wire technique under hyperemia. Murray law-based QFR (μQFR) was computed from invasive coronary angiography. The vertical height difference between the coronary ostium and the distal sensor position was measured on coronary angiographic images from a lateral view, and the hydrostatic pressure offset was added to the distal pressure reading. FFR was recalculated with this hydrostatic correction. We compared uncorrected FFR and hydrostatic pressure-corrected FFR against μQFR.

**Results:**

Hydrostatic correction increased the mean FFR from 0.78 ± 0.11 to 0.81 ± 0.10 (*p* < 0.0001), virtually matching the mean μQFR (0.81 ± 0.10). The Pearson correlation between μQFR and FFR was high both before and after correction (*r* = 0.95, *p* < 0.0001). However, Bland–Altman analysis showed that correcting the hydrostatic error eliminated the small bias between FFR and μQFR (mean difference bias improved from +0.03 to 0.00). By ROC analysis, an uncorrected FFR <0.80 was best predicted by μQFR <0.84 [area under the curve (AUC) 0.976], whereas using hydrostatic pressure-corrected FFR <0.80 corresponded to a μQFR threshold <0.78 (AUC 0.977).

**Conclusions:**

Correcting FFR for hydrostatic pressure improved its agreement with μQFR, suggesting that accounting for this error may enhance the consistency of physiologic lesion assessments.

## Introduction

Fractional flow reserve (FFR) measured during invasive coronary angiography is a well-established index of lesion-specific ischemia. FFR-guided percutaneous coronary intervention (PCI) has been shown to improve clinical outcomes by more accurately identifying flow-limiting stenoses compared to angiographic imaging alone (1–3). However, adoption of FFR in routine practice has been limited, in part due to the need for crossing the coronary stenosis by the pressure wire, administering hyperemic agents, added procedural time and cost. To overcome these limitations, several angiography-derived techniques for “functional coronary angiography” have emerged. Among these, quantitative flow ratio (QFR) has gained prominence for its high accuracy and robust evidence base (4, 5). QFR computes an FFR-equivalent value from routine angiographic images by 3D vessel reconstruction and fluid dynamics modeling, obviating the need for a pressure wire and pharmacologic hyperemia. A further refinement, the Murray law–based μQFR, implements artificial intelligence-assisted reconstruction of both the main branch and its side branches by applying principles of optimal vessel tapering (Murray's law) to define the true reference vessel diameter. This method enables QFR computation from a single angiographic projection of sufficient quality, reducing the time required for physiological lesion assessment (6, 7).

Most of the validation studies have demonstrated that angiography-derived (μ)QFR closely approximates invasive FFR (4–8). However, recent studies detected some systematic differences between (μ)QFR and invasive FFR both before and after the percutaneous coronary interventions (PCI) (9, 10).

One possible source of discrepancy between angiographic and invasive measurements is the hydrostatic pressure error during invasive pressure-wire FFR measurement. When a coronary pressure transducer is positioned at a different height than the arterial pressure reference (i.e., the tip of the guiding catheter at the ostium of the coronary), the gravitational hydrostatic pressure difference artificially skews the recorded distal pressure (Pd). In a supine patient, the distal LAD often lies higher than the aortic root, resulting in a lower measured Pd (and thus lower FFR) than the hypothetical value that would be detected if it was at the same level as the aortic pressure measurement (11–17).

Notably, angiographic QFR is inherently free from hydrostatic pressure bias—it estimates pressure drop from fluid dynamic principles and images, not from a sensor influenced by gravity. Thus, we hypothesized that part of the small systematic difference occasionally observed between QFR and invasive FFR in the LAD could be due to uncorrected hydrostatic pressure lowering the invasive FFR.

In this study, we aimed to quantify the impact of hydrostatic correction on LAD FFR measurements and determine if this correction improves the agreement between invasive FFR and μQFR. By doing so, we seek to refine the understanding of the potential discordance between FFR–QFR and its clinical relevance.

## Methods

### Study design and population

The present study constitutes a *post hoc* single-center observational substudy of the “Ready Register” (NCT04857762), which was a prospective, multicenter registry of patients who underwent invasive intracoronary FFR and RFR measurement comparing the visual estimate of coronary lesions and the functional severity of the stenosis assessed by invasive physiology measurements. The design of the “Ready Register” has been described in detail elsewhere (18). The inclusion criteria contained written, informed consent from all patients for the enrolment of their details into the database of the Registry for potential future analysis. Patients with CCS, who required functional intracoronary assessment with pressure guidewires, were eligible to enroll if the stenosis in one main coronary branch was assessed to be in the 40%–90% range of diameter reduction on the invasive coronary angiography. Exclusion criteria involved acute coronary syndrome, left main disease, contraindication for adenosine, coronary artery bypass graft on the investigated vessel, severe renal insufficiency (estimated glomerular filtration rate <30 mL/min/1.73 m^2^), and any medical comorbidity resulting in a life expectancy <12 months.

The main objective of this present substudy is to analyze paired invasive FFR and angiographic μQFR measurements in coronary lesions of the LAD. Because the hydrostatic pressure offset can be waited most pronouncedly when the sensor is positioned in the distal LAD, only LAD cases were included in this substudy. From 68 consecutive patients of one center (Nyíregyháza) in the Register, 38 LAD investigations were screened, and 33 fulfilled the strict angiographic criteria for this substudy. Only cases with a sharp contour of the LAD on the “key image” without obvious foreshortening were included in this study. We also excluded patients with significant overlapping branches, severe diffuse disease, or heavy calcification.

### Invasive coronary angiography and FFR measurement

Coronary angiography was performed by standard techniques. FFR was measured using a pressure guidewire (PressureWire X—Abbott, Santa Clara, CA, USA) introduced through a 6F guiding catheter engaged in the left main ostium. The guiding catheter was zeroed and normalized to aortic pressure at the ostium. The sensor of the wire was advanced to at least 2 cm distal to the stenosis, preferably to the distal segment. At the distal position, mean coronary pressure (Pd) and aortic pressure (Pa) were recorded, and resting Pd/Pa was determined. Hyperemia was induced by intracoronary adenosine (200 μg bolus) to achieve maximal hyperemia. FFR was calculated as the ratio Pd/Pa during hyperemia. After FFR measurement, the pressure wire was pulled back to ensure no drift in calibration (as there was no drift of >2 mmHg, no repeated measurement was performed in this cohort).

### μQFR analysis

**Figure 1:**
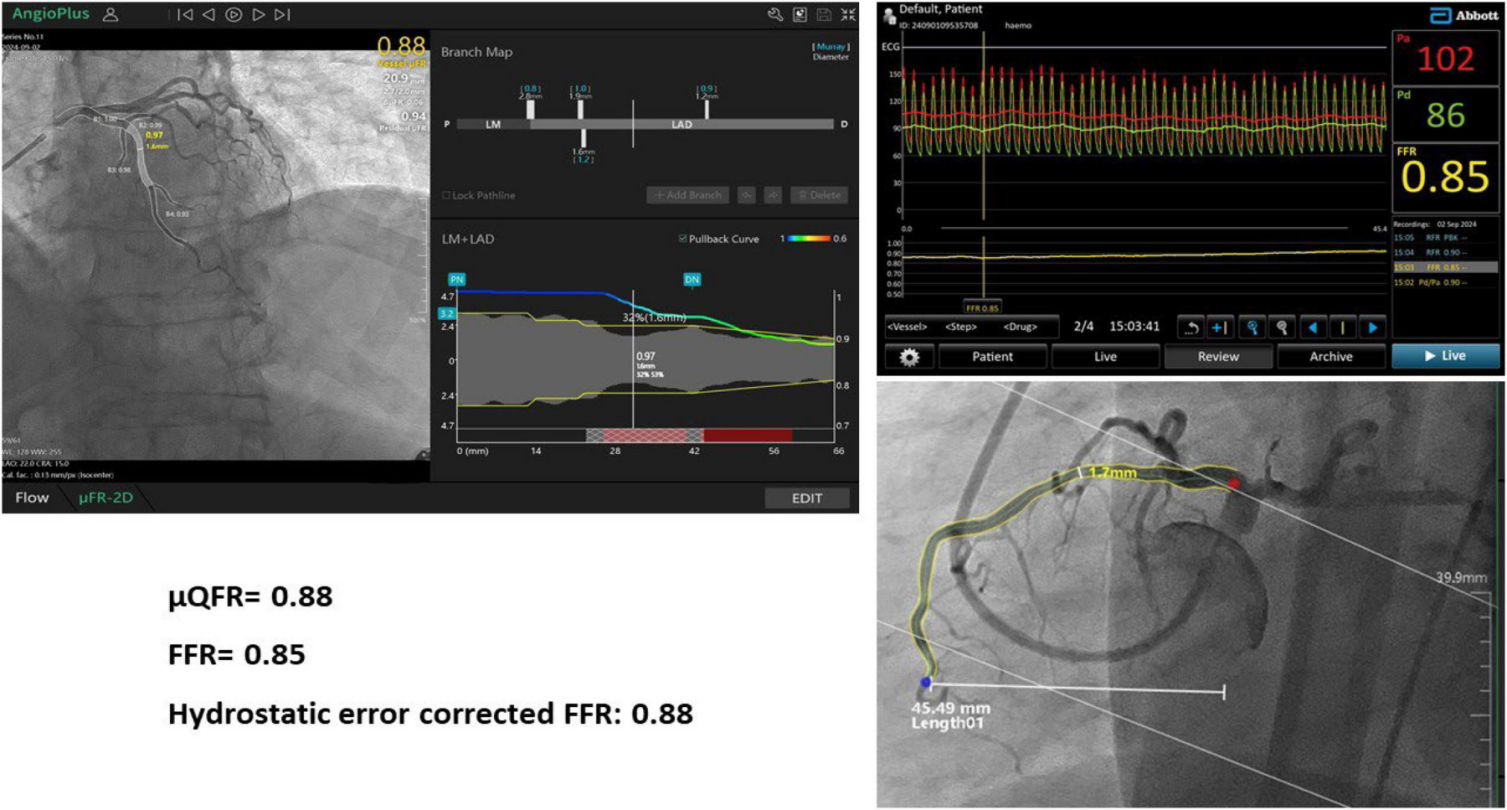
μQFR calculation and invasive FFR measurement without and with hydrostatic error correction. μQFR calculation showed 0.88 value (left upper panel), while invasive FFR indicated 0.85 (right upper panel). The right lower panel demonstrates the determination of the height difference between the site of the aortic pressure measurement and the sensor position from the lateral projection. The height difference results in 4.55 × 0.77 = 3.5 mmHg hydrostatic pressure. After adding this hydrostatic pressure to the distal pressure (86 + 3.5 = 89.5 mmHg), the FFR will be equal to the μQFR (0.88).

μQFR computation was performed offline using a validated software (AngioPlus Core, version V3,- Pulse Medical, Shanghai, China) by analysts blinded to the FFR results. μQFR specifically utilizes a single carefully chosen projection to derive the vessel's longitudinal profile, applying Murray's law to estimate the reference lumen diameter along the length of the vessel. μQFR was calculated for the hyperemic condition (extrapolated the contrast flow velocity from the resting frame count on the basis of previous databases) to yield an FFR-equivalent value. The μQFR value of the lesion was taken at the distal endpoint corresponding to the pressure wire sensor location (Figure 1). Side branches served as anatomical landmarks to facilitate co-registration between the sensor position (identified from lateral angiographic views) and the corresponding location on the μQFR “key image.”

### Hydrostatic pressure difference measurement and FFR correction

To quantify the hydrostatic pressure gradient between the coronary ostium and the distal LAD sensor position, we utilized the angiographic images. The height difference (vertical displacement) between the sensor position in the distal LAD and the catheter tip at the ostium was measured after automated calibration by the integrated distance measurement software of the x-ray system (Canon Alphenix Core+, Canon Medical Systems Europe B.V., The Netherlands).

The hydrostatic pressure offset was calculated as ΔP (mmHg) = height difference (cm) × 0.77 mmHg/cm, reflecting the density of blood (1.06 g/mL) in a gravitational field (acceleration 980 cm/s^2^). The invasive FFR was then corrected by adding the ΔP to the distal pressure for elevations (LAD), effectively removing the hydrostatic artifact. Thus, FFR_corrected_ = (Pd + ΔP)/Pa for LAD lesions (Figure 1).

### Statistical analysis

Continuous data are presented as mean ± standard deviation. Paired comparisons of FFR values before vs. after hydrostatic correction were made using a paired *t*-test. Statistical significance was defined by a two-tailed *p* < 0.05. Correlation between μQFR and FFR values was assessed by Pearson's correlation coefficient (*r*). Agreement between methods was further examined via Bland–Altman analysis, calculating the mean difference (bias) and 95% limits of agreement (±1.96 SD of the difference). To evaluate the diagnostic power of μQFR, we performed ROC curve analysis using invasive FFR ≤0.80 as the reference standard for ischemia. The optimal μQFR cut-off to predict FFR ≤0.80 was determined by the Youden index. AUC values for predicting FFR ≤0.80 by μQFR were calculated for both uncorrected and hydrostatic pressure-corrected FFR as reference. Analyses were conducted using MedCalc (MedCalc Software, Ostend, Belgium). Because this study was intended as a proof-of-concept analysis, no formal sample size calculation was performed; the sample size (33 vessels) was limited by the availability of cases with high-quality angiograms and pressure recordings suitable for precise hydrostatic measurement.

## Results

### Patient and lesion characteristics

The mean age of the 33 patients was 66.4 + 9.7 years; 67% were male; the frequencies of diabetes and hypertension were 36% and 85%, respectively (Table 1, Patient Characteristics). All lesions were in the LAD (by design); 12 (36%) were mid-LAD and 21 (64%) were distal LAD. The angiographic diameter stenosis averaged 64% ± 9%. All cases had TIMI 3 flow in the investigated coronary artery. The average aortic pressure during FFR was 97.02 ± 9.4 mmHg. The mean vertical distance from the ostium to the distal sensor was 4.38 ± 1.45 cm above the ostial level, corresponding to an estimated hydrostatic pressure difference of 3.37 ± 1.12 mmHg between the sensor and the catheter tip (range 0.85–6.46 mmHg). This magnitude of hydrostatic artifact is expected to reduce FFR by 0.03 ± 0.01 (up to 0.06 in the tallest cases) if uncorrected.

**Table 1:** Patient characteristics.

	All	Males	Females
Number	33	9	24
Age	64.36 ± 10.00	64.71 ± 9.48	63.44 ± 11.83
EF%	56.42 ± 13.34	53.29 ± 13.94	64.78 ± 6.78
Hypertension	31	22	9
Diabetes	12	9	3
MLD (mm)	1.84 ± 0.50	1.80 ± 0.41	1.96 ± 0.74
FFR	0.78 ± 0.11	0.74 ± 0.11	0.86 ± 0.04
μQFR	0.81 ± 0.1	0.78 ± 0.1	0.88 ± 0.04
Vertical distance	−4.38 ± 1.47	−4.83 ± 1.35	−3.18 ± 1.1

### FFR vs. μQFR before and after hydrostatic pressure correction

The invasive FFR values prior to correction ranged from 0.53 to 0.95, with a mean of 0.78 ± 0.11. The μQFR values ranged from 0.62 to 0.94, with a mean of 0.81 ± 0.10, resulting in a mean difference of +0.03 in the QFR-FFR value. After applying hydrostatic correction, the recalculated FFR values ranged from 0.57 to 0.97, mean of 0.81 ± 0.10.

### Correlation and agreement

**Figure 2:**
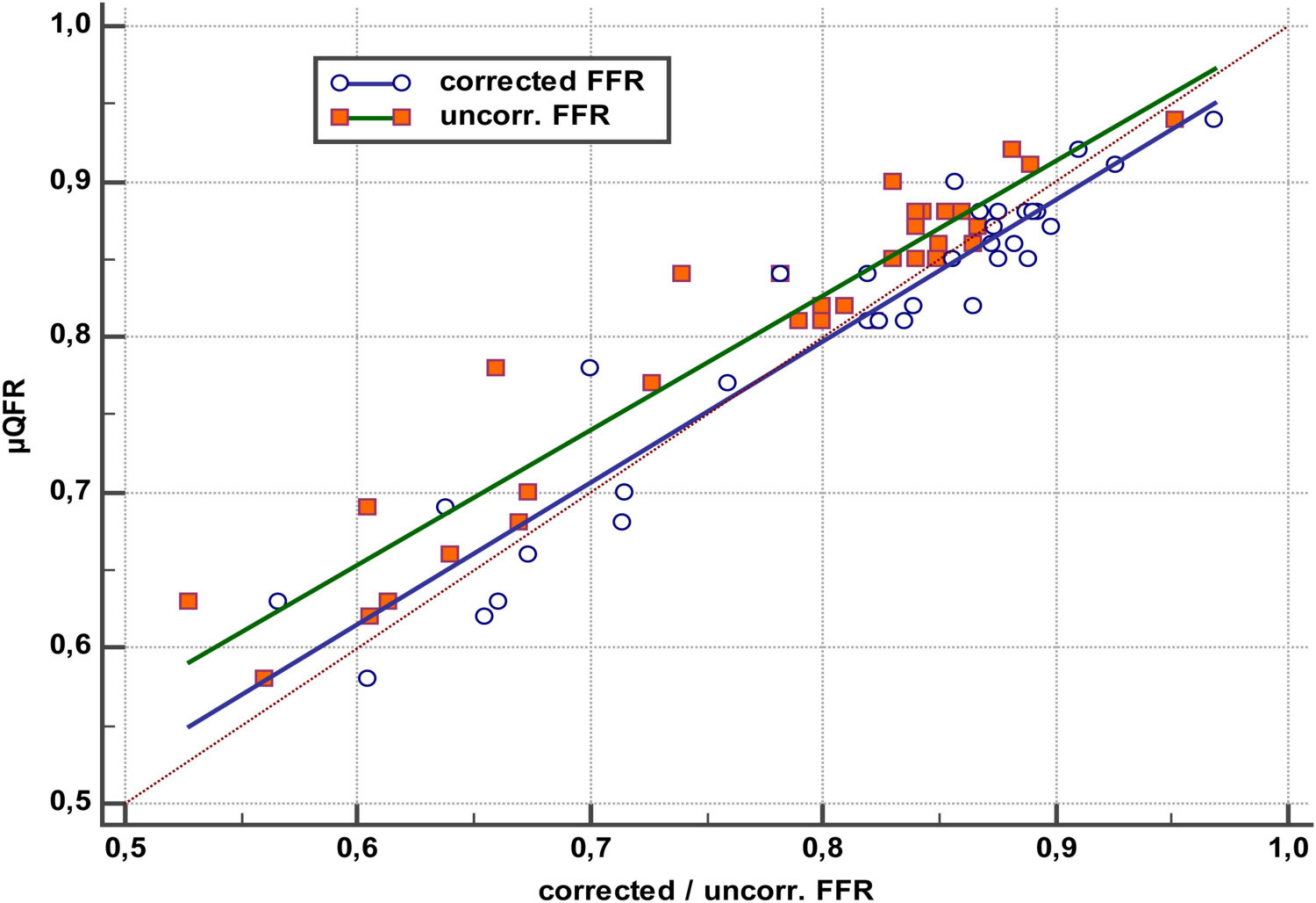
Correlation (and linear regression) scatter plots of μQFR vs. FFR without and with hydrostatic pressure correction. In the upper scatter plots, the red squares represent the measured invasive FFR values without hydrostatic pressure correction, and the blue circles with the correction. The fitted linear regression curves without and with correction are shown in green and blue, respectively.

**Figure 3:**
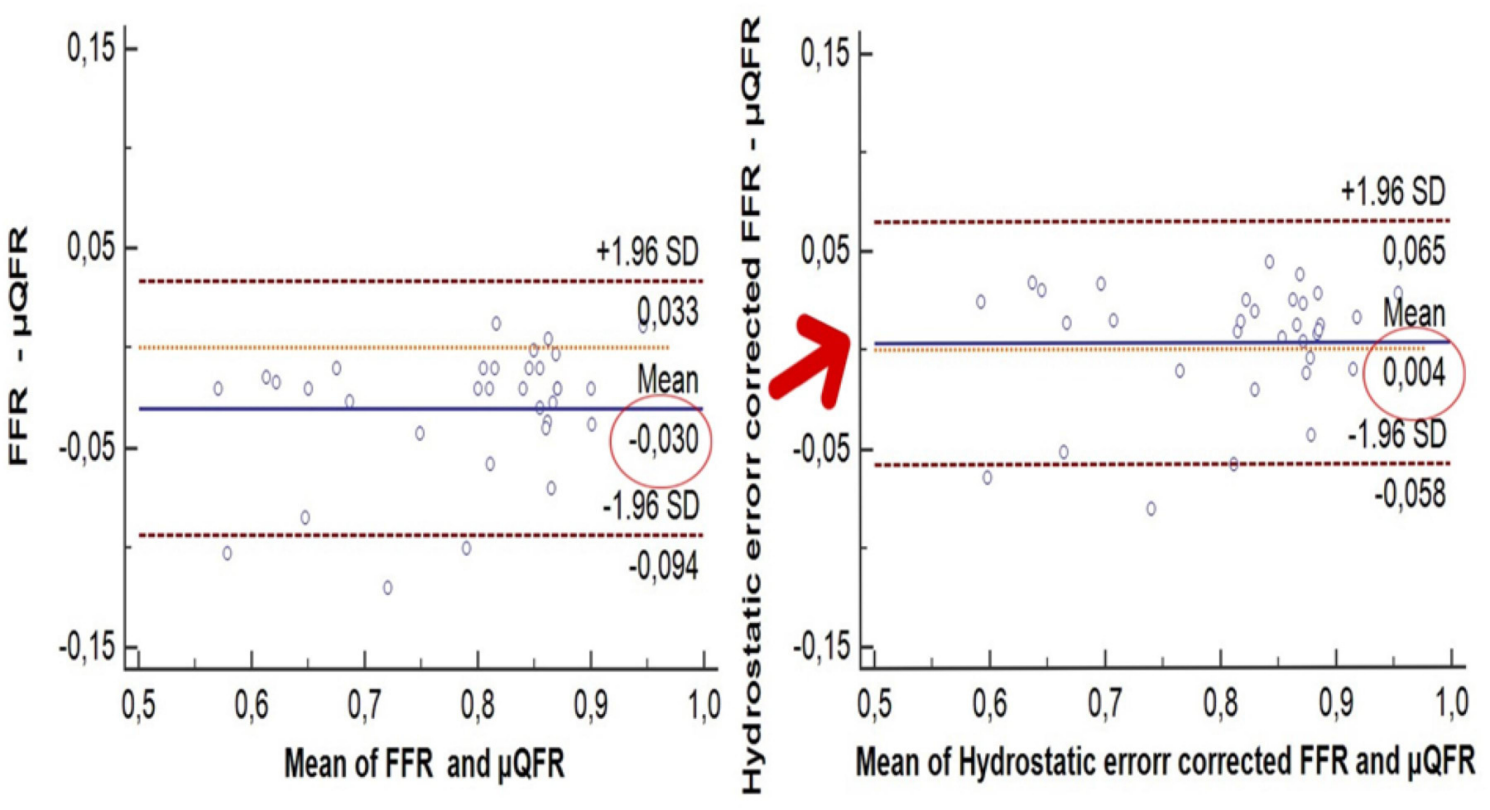
Bland–Altman plots of FFR vs. μQFR before (left) and after hydrostatic pressure correction (right) the scatter plot of μQFR vs. FFR points shifts upward with correction; clustering along the identity.

μQFR and invasive FFR demonstrated good correlation in this cohort, both before and after hydrostatic pressure adjustment (Figure 2). The Pearson correlation coefficient between μQFR and uncorrected FFR was *r* = 0.956 (*p* < 0.0001), and between μQFR and corrected FFR was *r* = 0.954 (*p* < 0.0001). Thus, the linear correlation was essentially unchanged by the correction (reflecting that hydrostatic error adds a nearly constant bias in one direction). However, Bland–Altman analysis revealed a clear improvement in agreement after correction (Figure 3). For uncorrected FFR vs. μQFR, the mean difference (μQFR—FFR) was +0.03 with 95% limits of agreement from −0.09 to +0.03. After hydrostatic pressure correction, the mean difference decreased to 0.00, and the 95% limits of agreement were −0.06 to +0.07. No significant proportional bias was observed in Bland–Altman plots.

### Threshold analysis (ROC for ischemia)

**Figure 4:**
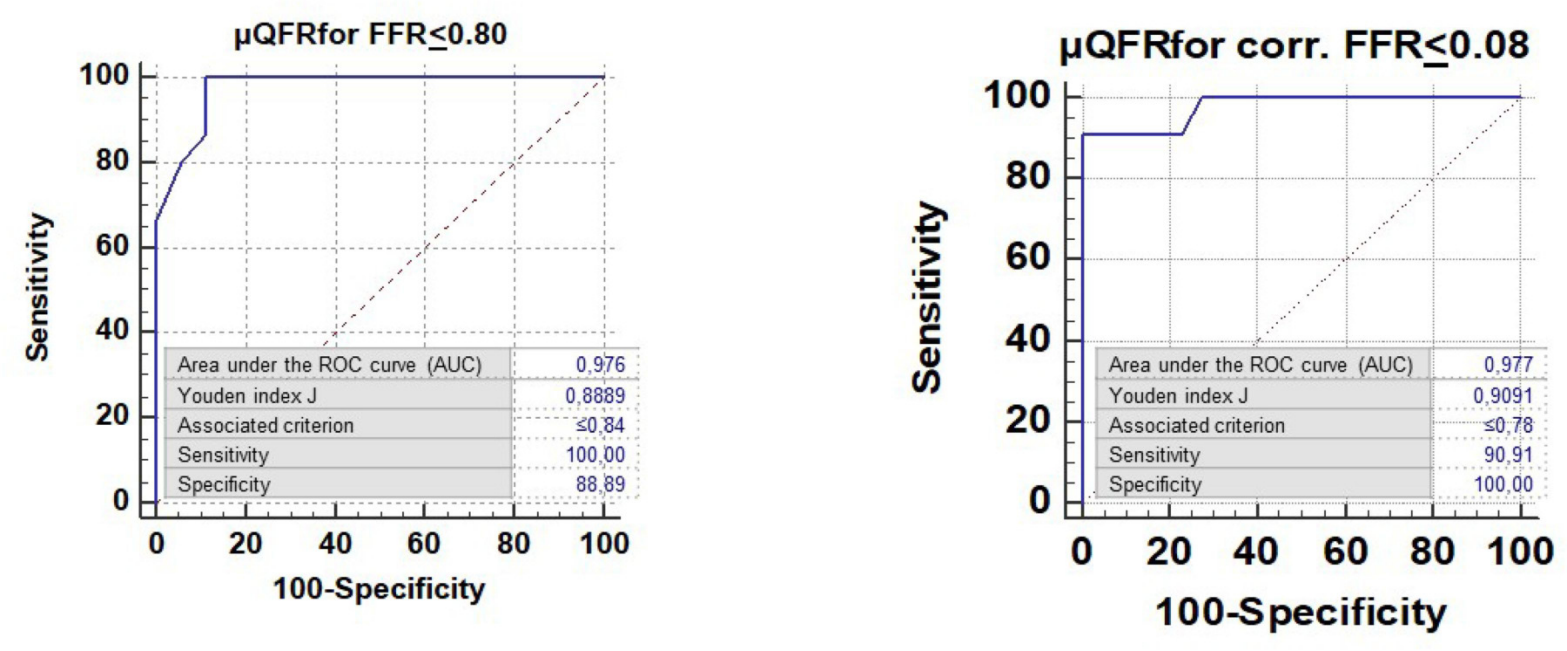
ROC analysis for ischemia.

We examined how well μQFR could predict whether a lesion's invasive FFR was ≤0.80, and whether the optimal QFR threshold differed when using hydrostatic-corrected FFR vs. uncorrected. For the uncorrected FFR as reference, the ROC curve for μQFR predicting FFR ≤ 0.80 had an AUC of 0.976, indicating outstanding diagnostic power. The optimal μQFR threshold was 0.84, with a sensitivity of 100% and a specificity of 89%. In contrast, when using the hydrostatically corrected FFR values as the reference standard, μQFR's AUC was 0.977, and the optimal cut-off of μQFR was ≤0.78 for predicting corrected FFR ≤0.80 with a sensitivity of 91% and a specificity of 100%. (Figure 4, Table 2).

**Table 2:** Quantitative comparison of the diagnostic values of μQFR and invasive FFR in LAD lesions (*n* = 33).

Metric	Without hydrostatic correction	With hydrostatic correction	*p*:
Mean FFR ± SD	0.78 ± 0.11	0.81 ± 0.10	*p* < 0.0001
Pearson correlation (μQFR vs. FFR), *r*	0.956	0.954	ns
Bland–Altman bias (μQFR—FFR)	+0.03	+0.00	na
Bland–Altman level of agreement (LOA)	−0.08 to +0.14	−0.09 to +0.09	ns
ROC AUC for μQFR predicting FFR ≤ 0.80	0.976	0.977	ns
Youden index	0.89	0.91	ns
Optimal μQFR threshold for FFR ≤ 0.80	≤0.84	≤0.78	na
Sensitivity at optimal threshold (%)	100%	91%	na
Specificity at optimal threshold (%)	89%	100%	na
Diagnostic accuracy with 0.80 μQFR predicting FFR ≤ 0.80	85%	97%	na
Diagnostic accuracy with specific μQFR values (0.84, 0.78) predicting FFR ≤ 0.80	94%	97%	na

FFR, fractional flow reserve; μQFR, Murray law-based quantitative flow ratio; SD, standard deviation; LOA, limits of agreement; ROC AUC, area under the receiver-operating characteristic curve. ns, non-significant; na, not applicable.

In Table 2 the main diagnostic parameters and values found before and after hydrostatic pressure correction are summarized.

### Additional findings

**Figure 5:**
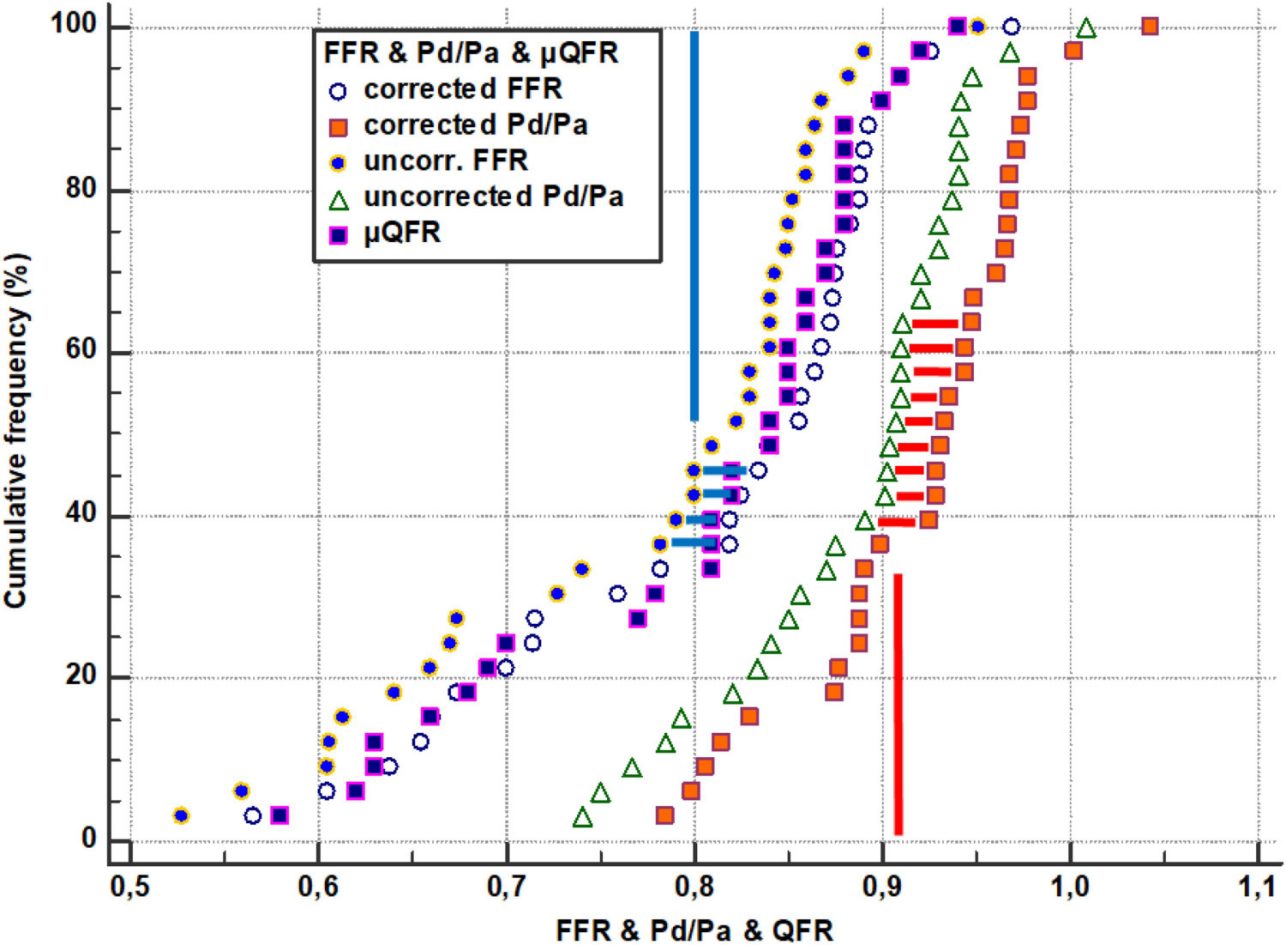
FFR and Pd/Pa with and without hydrostatic pressure correction, and the μQFR values. Cumulative frequencies of the corrected and uncorrected FFR and Pd/Pa, as well as the μQFR values. The blue vertical line indicates the 0.80 cut-off value for the FFR, and the red vertical line represents the generally accepted resting 0.91 Pd/Pa cut-off. After hydrostatic pressure correction, the FFR values (uncorrected: blue circles, corrected: empty blue circles) got very close to the μQFR distributions (blue squares), and 4 cases rose above the 0.80 FFR cut-off (short horizontal lines). The resting Pd/Pa (uncorrected: green triangles, corrected: red squares) increased to a non-ischemic range >0.91 (short red lines) in 9 cases.

In this limited sample, 11 lesions had an uncorrected FFR ≤0.80 (ischemic by standard criteria). After hydrostatic correction, 4 out of those 11 rose to above the cut-off, theoretically changing classification to non-ischemic (Figure 5). Thus, hydrostatic adjustment reclassified 4 of 33 lesions (12%) from FFR-positive to FFR-negative relative to the 0.80 cut-off, consistent with expectations that a small subset of borderline LAD lesions may shift above the threshold when physiological values without hydrostatic pressure are considered. For those 4 lesions that moved through the cut off (FFR-positive to negative), the μQFR values were 0.81 and 0.84,—in retrospect indicating a mismatch. This illustrates a tangible clinical example of how μQFR and FFR disagreement in borderline LAD cases can sometimes be explained by uncorrected hydrostatic pressure artifacts.

## Discussion

Outcome studies have demonstrated both the clinical and economic benefits of a physiology-guided revascularization strategy in chronic coronary syndromes, whereby only ischemia-producing lesions are treated. Invasive fractional flow reserve (FFR) measurement with a pressure wire has been established as the gold standard for identifying lesion-specific ischemia (1–3).

However, this technique has an inherent limitation in accurately distinguishing the pressure loss caused by the stenosis itself from the hydrostatic pressure difference between the proximal and distal pressure measurements when a substantial vertical height difference exists between the pressure sensor and the guiding catheter tip.

The hydrostatic component alters the measured distal pressure by approximately 0.77 mmHg for every centimeter of height difference (13). Because hydrostatic pressure affects both the coronary arterial and venous systems equally, it does not contribute to the effective coronary perfusion pressure and therefore does not influence coronary blood flow. Consequently, hydrostatic pressure should be regarded as a measurement artifact rather than a physiologically relevant pressure loss when assessing the ischemic significance of a coronary stenosis (11–17).

In contrast, angiography-derived FFR, often referred to as “functional coronary angiography,” is inherently free from this limitation. The pressure gradient is estimated using hydrodynamic principles, based on anatomical characteristics of the coronary artery and assumptions about coronary flow velocity, with no contribution from hydrostatic pressure (4–7). As a result, angiography-derived FFR reflects the true flow-limiting effect of the coronary lesion without contamination by gravity-induced pressure differences.

In this study, we examined the agreement between angiography-derived μQFR and invasively measured FFR in LAD coronary lesions, with a particular focus on the impact of hydrostatic pressure correction on FFR. We found that correcting for the hydrostatic pressure gradient significantly improved the level of agreement between the μQFR and the corrected FFR values in the Bland–Altman analysis compared to the uncorrected ones. Before correction, μQFR values tended to be slightly higher than pressure-wire FFR (mean difference 0.03).

In our series with 33 cases with borderline LAD lesions, 4 (12%) became negative after correction, highlighting that the hydrostatic pressure correction near the cut-off value of the FFR potentially could influence the decision about the PCI. Our findings are consistent with previous ones showing that correcting for hydrostatic pressure would change clinical FFR classification (ischemic vs. non-ischemic) in up to 8%–12% of cases for the LAD, in patients with coronary stenosis with intermediate severity (11–13).

These results can challenge the application of the universal 0.80 FFR cut-off for the indication of revascularization, despite the generally accepted application of a single pre-intervention FFR threshold for all coronary arteries.

On the other hand, several published data from the follow-up of the post-stent populations showed that the prognostic cut-off value of the post-stent FFR should be evaluated in vessel vessel-specific way. These data have been summarized in a meta-analysis of Collet et al.: the prognostic cut-off value of FFR predicting target vessel failure after stent implantation was different in the left anterior descending artery (LAD) and the non-LAD arteries (19). The average difference in their cohort “*between LAD and non-LAD vessels was 0.064, consistent with the expectation regarding hydrostatic effects (LAD 0.04 lower from a 5-cm elevation, LCX or RCA 0.02 higher because of a 3- to 4-cm depression; net difference = 0.06*)”.

The controversy between the universally applied pre-stent prognostic value of the FFR for all coronary artery branches vs. a vessel-specific approach in the post-stent scenario might be explained by the inherent ranges of pre- and post-intervention FFR values. Before stenting, the average FFR values are considerably lower, and the scatter is generally much higher than after the stenting, with a higher average FFR. Therefore, in the pre-stent scenario, the relative impact of hydrostatic pressure errors near the diagnostic cutoff may have attenuated.

The seemingly different relevance of the hydrostatic effect on FFR before and after stenting is resonant of the different impact of the hydrostatic effect on the binary cut-off of the pressure ratios during resting state (Pd/Pa, iFR) and hyperemia (FFR) (20). Approximately one-third of the resting pressure ratios and less than one-fourth of FFR were re-classified across an ischemic threshold due to the consideration of the hydrostatic pressure in the previously analyzed patient populations dominated by LAD cases (11–17).

In our present study, we also examined the reclassification rate after hydrostatic pressure correction using the 0.91 cut-off value for the resting Pd/Pa, and we found 9/33 (27%) cases, in contrast to the much lower 4/33 (12%) reclassification rate for FFR (Figure 5). It could be concluded that the hydrostatic effect demonstrates less impact in the distribution of the higher pressure ratios, like in the cases of the resting Pd/Pa and the post-PCI FFR, than in the lower range of the values of the pre-PCI FFR.

Recent studies detected some systematic differences between the (μ)QFR and the invasive FFR, both before and after the percutaneous coronary interventions (PCI). Namely, Wu et al. published a 0.02 and 0.03 “overestimations” of the QFR compared to invasive FFR before and after the PCI, respectively (9), while Csanádi et al. detected a slightly higher mean bias of 0.048 and 0.052 between pre-PCI and post-PCI QFR vs. FFR, respectively (10), in their patient population dominated by LAD cases. Similarly, in our cohort, 9 uncorrected resting Pd/Pa ratios increased from below to above the cut-off value as opposed to only 4 such FFR values.

In our study, the average difference of −0.03 between the post-PCI FFR and μQFR is consistent with the above data, but this is in contrast to the results of the FAVOR III Europe trial (21). In that study, the median QFR value in the group of QFR-guided revascularization was lower than that of the FFR in the group randomized to invasive FFR guidance. However, as it was discussed extensively, the results of the FAVOR III Europe trial can associate “*…the poorer performance of QFR, with too many clinically relevant false negatives in a clinical, multicentre setting, and the reliance on in-procedure QFR analysis*” (22).

In a broader perspective, the high degree of agreement between μQFR and FFR adds to the growing validation of angiographic flow indices as practical alternatives to wire-based physiology. We observed an overall Pearson correlation of about 0.95, and ROC AUC 0.97 for μQFR vs. FFR, which are in line with or even slightly better than the figures reported in multicenter QFR validation studies (4–9). This likely reflects the controlled conditions of our analysis (all LAD lesions, high-quality angiograms, careful methodology).

We confirmed that the hydrostatic effect can lead to a systematic underestimation of “true” FFR in the LAD. While the clinical impact of hydrostatic correction prior to revascularization has not been established in large outcome studies, emerging post-PCI data suggest that even small physiological differences may influence management. The true physiological resistance of the LAD may be better reflected by higher FFR values after correction for hydrostatic pressure. In reclassified borderline cases, clinical decision-making should remain individualized. Importantly, pullback measurements assessing lesion focality should guide revascularization decisions. In the absence of a well-defined focal pressure drop, the benefit of stenting is uncertain, and accounting for hydrostatic pressure may further support deferral of intervention in borderline LAD lesions.

### Limitations

This *post hoc* substudy is limited by its small sample size and single-center design, including only 33 LAD lesions selected based on strict angiographic criteria. These factors substantially limit the generalizability of our findings and introduce potential selection bias.

Our hydrostatic measurement method, while based on angiographic imaging, may carry some measurement errors (we estimated height differences via imaging; slight angulation or calibration errors could introduce a millimeter or two of uncertainty). In a previous study (11), we compared 2D height measurements obtained from lateral angiographic views using x-ray quantification software with 3D reconstruction-based calculations derived from diastolic frames using two projections. A close correlation was observed between the two approaches in 41 coronary vessels from 37 patients. However, we acknowledged that the accuracy of 3D reconstruction itself depends on the selection of minimally foreshortened projections with at least a 25° angular separation, which also represents a methodological limitation.

Only LAD cases were included in this substudy, because the hydrostatic pressure has the largest effect in this vessel. In the LCx the opposite direction of the height difference results an opposite effect on the measured distal pressure, which may explain that the hydrostatic effect could be neutralized in an average population. It could underline the need for further exploration of the vessel specific physiological analyses.

Another point is that μQFR calculation in this study used extrapolated hyperemic flow from the resting frame count. The extrapolation is based on a database from a previous study and does not necessarily represent the individual hyperemic flow, also determined by the microvascular capacity (23). It would be interesting for future research to evaluate the hyperemic frame count and its relation to the real blood flow velocity, preferably together with established flow determination like Doppler or continuous thermodilution technique. Obviously, it would be possible to use machine learning techniques on a teaching database for a reliable algorithm of automatic determination of hyperemic flow velocity. Theoretically, the hyperemic angiography could provide more exact velocity data for pressure gradient calculation, and could provide a platform for the assessment of the microvascular function. Finally, this study was restricted to comparing the angio-based and the invasive physiological investigations, but clinical outcomes were not tracked. Nevertheless, by bridging an important technical gap, our findings can contribute to improving the accuracy of physiologic lesion assessment, which ultimately could translate to better patient selection for revascularization (24).

## Conclusion

Angiography-derived quantitative flow ratio (μQFR) shows a good correlation and agreement with invasive FFR for assessing LAD coronary stenosis, but a systematic underestimation of FFR in the distal LAD due to hydrostatic pressure can explain a minor discrepancy between the two methods. Correcting the invasive FFR for hydrostatic pressure increased FFR values by 0.03 on average, eliminating the bias and yielding practically identical mean values to μQFR.

Further investigation in larger patient populations, both in native coronary lesions and post-stent settings, is warranted to better define the role of hydrostatic pressure correction in invasive physiological assessment, its agreement with functional coronary angiography, and its potential clinical relevance.

## Data Availability

The raw data supporting the conclusions of this article will be made available by the authors, without undue reservation.
